# The “Local Neighborhood” Effect of Environmental Regulation on Green Innovation Efficiency: Evidence from China

**DOI:** 10.3390/ijerph191610389

**Published:** 2022-08-20

**Authors:** Yanwei Lyu, Jinning Zhang, Fei Yang, Di Wu

**Affiliations:** School of Business, Shandong University, Weihai 264209, China or

**Keywords:** environmental regulation, green innovation efficiency, “local neighborhood” effect, transfer of polluting industries

## Abstract

Current research has generally concentrated on the motivations of environmental policies on local green innovation while ignoring the effect they may have on green innovation in neighboring places. To obtain a thorough understanding and explanation of the influencing mechanism of environmental regulation (ER) on green innovation efficiency (GIE), the super-slack based measure-data envelopment analysis (Super-SBM-DEA) method was applied to evaluate Chinese provinces’ GIE, a spatial Durbin model was developed to evaluate the effect of ER on GIE from the perspective of the “local neighborhood” effect, and a mediating effect model was built to analyze the transmission mechanism of the neighborhood effect of ER on GIE. The study indicated that China’s regional GIE is high in the east and low in the west, with large spatial variability and significant positive spatial clustering characteristics. The effect of ER on local GIE is “U” shaped, while the influence on green innovation efficiency in neighboring areas is an inverted “U” shape. The influence of environmental regulation on GIE in neighboring areas is mainly achieved through the transfer of local polluting industries to neighboring areas. Based on the results, policy recommendations from the perspectives of choosing environmental regulation tools and transferring polluting industries are made to promote and realize the coordinated development of ER and green innovation.

## 1. Introduction

China’s economy has experienced leapfrog growth over the past several decades; however, environmental problems have become increasingly prominent [1]. According to the *2020 Global Environmental Performance Index* (EPI) *Report*, jointly published by Yale University and other research institutions, China ranked 120th out of 180 participating countries and regions worldwide, with a score of 37.3, reflecting China’s poor environmental performance on the global assessment scale. In particular, China is at the bottom in terms of air quality, biodiversity and climate change. An indisputable problem is that resource constraints, environmental degradation and increased haze have constrained China’s sustainable per capita gross domestic product (PGDP) while also increasing uncertainty about global development and governance [2,3]. In response to severe environmental problems and challenges, the Chinese government has proposed the strategic deployment of “Promoting green development and accelerating the establishment of ecological civilization” in the report of the 19th National Congress, and the 14th Five-Year Plan further clarifies that it is compulsory to support green technology innovation and promote cleaner production. Strengthening environmental governance and promoting green and sustainable development have become a consensus in society [4,5]. In the same manner, the government of China has promulgated and implemented several environmental management policies and initiatives and has gradually strengthened environmental regulations with a view to enhancing environmental performance and achieving green development. However, strict environmental regulation measures in the short term may, for one thing, increase the cost of pollution treatment for companies, crowd out research and development (R&D) funds and adversely affect technological innovation [6,7]. In addition, it also tends to stimulate the short-sighted behavior of local governments, resulting in a game of economic growth and environmental protection between local and neighboring areas, which, in turn, leads to problems such as the transfer of polluting enterprises [8,9]. Over the long term, solving environmental pollution problems will inevitably rely on technological progress [10], particularly green technology-oriented innovation [11]. Green technology innovation is an important support for promoting high-quality economic development and achieving energy-saving and emission reduction targets. In 2019, China’s National Development and Reform Commission, together with the Ministry of Science and Technology, issued guidelines for building green technology innovation. The document points out that green technology innovation is increasingly becoming an important driving force for green development and an important support for fighting the battle against pollution, advancing ecological civilization and promoting high-quality development. Only by vigorously promoting green innovation and enhancing enterprises’ green innovation performance can we achieve the win-win goal of advancing the economy and the environment.

Scholars have conducted a wide range of research on the connection between ER and green innovation, resulting in the following four main views. First, on account of the classical Porter hypothesis, several scholars debate that reasonable ER can generate an “innovation compensation effect”, meaning that the costs of environmental regulation can be offset by the compensation effect of innovation [12]; thus, environmental regulation has been strengthened to be instrumental in green technological innovation [13]. Borghesi et al. [14] showed through micro evidence from the Italian manufacturing sector that environmental regulation is an essential driver of technological innovation, especially the development of clean technology. Based on provincial panel data in China, Song et al. [15] found that both formal and informal environmental regulations can promote technological innovation. Chen et al. [16] tested the existence of Porter’s hypothesis at the prefectural city level in China and found that a 1% increase in the intensity of environmental regulations will increase green technological innovation by 0.173%, and this finding still holds when replacing different indicators. Rubashkina et al. [17] verified the presence of the weak Porter hypothesis in European manufacturing firms mainly using an instrumental variable method, but found no evidence for the strong Porter hypothesis. Second, from the perspective of neoclassical economics, some academics contend that environmental regulations raise businesses’ costs, so regulation will always inhibit the green technology innovation activities of enterprises [18]. This view is simply summarized as the “compliance cost argument”. Chintrakam and Weber [19] found by investigating the manufacturing statistics of 48 states in the United States that the strengthening of environmental regulation will make the manufacturing industry inefficient, which may be due to a lack of sufficient funds for the development of environmental protection patents due to environmental regulation [20]. Kneller and Manderson [6] examine environmental regulation and creative activities in the UK manufacturing sector from 2000 to 2006 and show that ER can crowd out firms’ investment in innovation, particularly in industries with high environmental costs.

A third view is that ER has a nonlinear influence on technological innovation. Several scholars have found a “U”-shaped relationship between ER and green technology innovation [21]; in addition, an inverted “U”-shaped relationship has also been confirmed [22,23]. The fourth is the belief that the relationship between the two is uncertain [24]. The study by Amores-Salvadó et al. [25] points out that the influence of ER on technological innovation varies in the long and short term. Specifically, in the short run, ER can crowd out green technology R&D investments [26], but in the long term, the compensatory effects of innovation can produce extra profits to facilitate technological advances [27]. Some literature also explored the spatial role and spillover effects of ER on green innovation [28]. Fan et al. [29] found a significant positive correlation in green innovation efficiency among Chinese cities through a spatial autocorrelation test, and a remarkable spatial spillover effect of ER on green innovation. Zhou et al. [30] found not only a significant positive spatial spillover effect of ER on urban innovation based on panel data of 41 cities in the Yangtze River Delta of China, but also a negative spatial spillover effect of urban sprawl on innovation capacity of neighboring cities.

In summary, domestic and international scholars have conducted fruitful research on this issue, which provides a reference for the writing of this paper, but there are still the following areas to be expanded: first, most of the literature uses indicators, such as R&D expenditure or the number of green technology patents, to represent green innovation [31,32] and then examines whether ER is conducive to stimulating green innovation. Considering the definition of green innovation proposed by Kemp and Pontoglio [18], green innovation includes both knowledge technology and product innovation, and the employment of a single indicator is not sufficient to evaluate the real situation of green innovation. Second, most of the available literature treats the study area as a closed system, ignoring the spatial spillover effects of ER on the impact of green innovation [33,34]. Nevertheless, ER affects local green innovation and has spillover effects on green innovation in neighboring areas [35]. To more accurately describe the influence of environmental regulation on green innovation, it is necessary to further consider spatial factors.

The possible contributions and innovation of this paper are as follows. First, the paper fully considers the environmental constraints in the process of PGDP and constructs the Super-SBM-DEA model to evaluate each province’s GIE in China. Second, this study innovatively takes the perspective of “local-neighborhood” technological progress of environmental regulation and applies the spatial Durbin model to test it empirically. Third, based on the characteristic facts of regional industrial transfer, a mediating effect model is used to explore the mechanisms and influence channels of ER on GIE. This research not only effectively expands the theoretical connotation of the Porter hypothesis, but also helps to promote and realize the coordinated process of green technology innovation and interregional environmental regulations.

## 2. Mechanism Analysis and Hypothesis

### 2.1. Local Effect of ER on Green Innovation

The effect of ER on regional green innovation activities has two facets, namely, the positive “innovation compensation effect” and the negative “compliance cost effect”. Currently, the main means of government environmental regulation is to set standards for pollution emissions of enterprises or to purchase emission permits [36], both of which obviously increase the cost of treatment for enterprises [37]. In the short term, the cost of tackling pollution can crowd out investment in research and innovation, so ER is not instrumental in green technology innovation, thus creating a “compliance cost” effect [38]. In the long term, enterprises often choose to increase investment in green technology development and research, realize energy saving and emission reduction business models and improve production efficiency through technological innovation, resulting in an “innovation compensation” effect [39]. Environmental regulation acts as a disincentive when the “compliance cost” effect dominates and as a facilitator when the “innovation compensation” effect dominates.

Therefore, the relative size of the two effects determines the direction and size of the final effect. Further, in a static framework where the intensity of ER is low, the introduction of ER only increases the cost burden of enterprises, compresses their profit margins, and does not motivate them to undertake technological R&D to cover the current costs of pollution control in the short term [37], at which point the “compliance cost” effect dominates and has a negative impact on local green innovation. In contrast, as ER increases, firms in a dynamic economic framework will change the situation accordingly, and ER will make them tend to increase their technological R&D investments. This leads to an increase in productivity [40], which in turn offsets the negative impact of “compliance cost” effect. In this case, the “innovation compensation” effect is dominate and has a positive impact on local green innovation. Accordingly, Hypothesis 1 is proposed.

**Hypothesis** **1.** *The effect of ER on local green innovation is “U”-shaped: ER first inhibits and then promotes green innovation.*

### 2.2. Neighborhood Effects of ER on Green Innovation

There are also two sides to the influence of ER on neighboring green innovation activities. In the short term, when the intensity of local ER is high, local polluting industries may shift to neighboring regions. At this time, neighboring regions achieve higher income levels due to increased output [41], which, in turn, will increase the investment in green technology R&D, thus promoting green innovation in neighboring regions. However, in the long run, this situation will change. If the intensity of local ER increases and the ER of neighboring areas do not change in tandem with it, the neighboring areas tend to become the continuation of polluting industries [42]. Since most of the industries to be taken over are highly polluting, the industrial structure of the neighboring areas tends to develop in a non-clean direction, resulting in a “pollution haven effect” similar to that common in international investment [43], which ultimately inhibits green technology innovation in the neighboring areas. Based on this, Hypothesis 2 is proposed.

**Hypothesis** **2.** *The impact of ER on green innovation in neighboring areas is an inverted “U”-shape: ER promotes and then inhibits green innovation.*

## 3. Methods and Data

### 3.1. Model Construction

#### 3.1.1. Efficiency Measurement Model

Assume that the system has n decision-making units, each with input X, desired output $Y^{g}$ and nondesired output $Y^{b}$ vectors with elements $X\in R^{m}$, $Y^{g}\in R^{s_{1}}$ and $Y^{b}\in R^{s_{2}}$. The matrices X, $Y^{g}$ and $Y^{b}$ are defined as follows: $X=\left[x_{1},\ldots ,x_{2}\right]\in R^{m\times n}$, $Y^{g}=\left[y_{1}^{g},\ldots ,y_{n}^{g}\right]\in R^{s_{1}\times n}$, $Y^{b}=\left[y_{1}^{b},\ldots ,y_{n}^{b}\right]\in R^{s_{2}\times n}$, where, $x_{i}>0$, $y_{i}^{g}>0\;\mathrm{and}\;y_{i}^{b}>0\left(i=1,2,\ldots n\right)$. Therefore, the Super-SBM-DEA model can be expressed as:

$$\rho =\min\frac{\frac{1}{m}\sum_{i=1}^{m}\frac{{\bar{x}}_{i}}{x_{i0}}}{\frac{1}{s_{1}+s_{2}}(\sum_{r=1}^{s_{1}}\frac{{\bar{y}}_{r}^{g}}{y_{r0}^{g}}+\sum_{l=1}^{s_{2}}\frac{{\bar{y}}_{l}^{b}}{y_{l0}^{b}})}$$

(1) $$s.t\{\begin{array}{c} \bar{x}\geq \overset{n}{\underset{j=1,\neq 0}{\sum }}\lambda_{j}x_{j}\\ {\bar{y}}^{g}\leq \overset{n}{\underset{j=1,\neq 0}{\sum }}\lambda_{j}y_{j}^{g}\\ {\bar{y}}^{b}\leq \overset{n}{\underset{j=1,\neq 0}{\sum }}\lambda_{j}y_{j}^{b}\\ \bar{x}\geq x_{0},{\bar{y}}^{g}\leq y_{0}^{g},{\bar{y}}^{b}\geq y_{0}^{b},{\bar{y}}^{g}\geq 0,\lambda \geq 0 \end{array}$$

In Equation (1), ρ represents the superefficiency value, with larger values indicating higher efficiency. λ is the weight vector, and $\bar{x}$, ${\bar{y}}^{g}$ and ${\bar{y}}^{b}$ are the slacks for inputs, desired outputs and undesired outputs, respectively.

The use of the DEA window analysis method first requires the determination of the window width d. In this paper, we refer to the common practice in the literature and determine that d=3. The cumulative time of the study is T. Then, T−d+1 windows will be built while each decision-making unit will be evaluated d times at the kth window using Equation (1), accumulatively (T−d+1)d times per decision-making unit, and the arithmetic mean of each time point will be regarded as the efficiency rating for that decision-making unit for each year.

#### 3.1.2. Spatial Econometric Model

The use of spatial econometric models first requires testing whether the main variables are correlated spatially, and existing studies have typically used *Moran’s* index (*Moran’s I*) for testing [44], which is calculated as:

(2) $$\mathrm{Moran}’s\;I=\frac{\sum_{i=1}^{\;n}\sum_{j=1}^{n}W_{ij}\left(x_{i}-\bar{x}\right)\left(x_{j}-\bar{x}\right)}{S^{2}\sum_{i=1}^{\;n}\sum_{j=1}^{n}W_{ij}}$$

In Equation (2), $x_{i}$ denotes the observed value of region *i*, and $\bar{x}$ and $s^{2}$ are its mean and variance, respectively. $W_{ij}$ is the spatial weight matrix. The value of *Moran’s I* ranges from [−1,1]. The greater the index value trends to 1, the stronger the spatial positive correlation; the greater the index value trends to −1, the stronger the spatial negative correlation; the more the index value trends to 0, the more the data are randomly distributed in space and have no spatial correlation.

This paper centers on the “local neighborhood” effect of ER on green innovation, which requires consideration of the influence of local ER on green innovation in neighborhoods. This relationship can be measured by the spatial Durbin model [45,46], which is therefore chosen to explore the “local-neighborhood” effect of ER on GIE.

The baseline regression model is:

(3) $$GIE_{it}=\alpha +\beta_{1}ER_{it}+\beta_{2}ER_{it}^{2}+\lambda X_{it}+\varepsilon_{it}$$

The SDM is:

(4) $$GIE_{it}=\rho W_{ij}IE_{it}+\beta_{1}ER_{it}+\beta_{2}ER_{it}^{2}+\lambda X_{it}+\theta_{1}W_{ij}ER_{it}+\theta_{2}W_{ij}ER_{jt}^{2}+\gamma W_{ij}X_{it}+\varepsilon_{it}\;\;\;\;\;\;\;\;\;$$

In Equations (3) and (4), $GIE_{it}$ represents the GIE of each province, while $ER_{it}$ represents environmental regulation, and ρ is the spatial autoregressive coefficient. $X_{it}$ is the control variable, α is the intercept term and $\varepsilon_{it}$ is the random disturbance term. $W_{ij}$ is the element of the spatial weight matrix. In this paper, we mainly use two spatial weight matrices. W1 is the geographic adjacency matrix and W2 is the geographic distance matrix, the expressions of which are as follows.

(5) $$W_{ij}=\{\begin{array}{c} 1,\;\;\;\;\;\;\;\;\;\;\;\;\;region\;i\;and\;j\;are\;adjacent\\ 0,\;\;\;\;\;region\;i\;and\;j\;are\;not\;adjacent \end{array}$$

(6) $$W_{ij}=\{\begin{array}{c} 1/d_{ij},\;\;i\neq j\\ 0,\;\;\;i=j \end{array}$$

In the above equation, *i* and *j* represent different regions, respectively, and *d* is the geographic distance between region *i* and region *j*.

#### 3.1.3. Mediating Effects Model

The impact of environmental regulations on local GIE has been shown to have a “U”-shaped relationship, and the influence on green innovation efficiency in neighboring areas has been shown to have an inverted “U”-shaped relationship. One possible reason is the transfer of polluting industries caused by ER [47], which, in turn, affects green innovation efficiency in neighboring places. This transmission channel was, therefore, further analyzed by constructing a mediating effects model, which was constructed as shown below:

(7) $$\;\;GIE_{it}=\rho W_{ij}GIE_{it}+c_{1}ER_{it}+c_{2}ER_{it}^{2}+\lambda X_{it}+c_{3}W_{ij}ER_{it}+c_{4}W_{ij}ER_{jt}^{2}+\gamma W_{ij}X_{it}+\varepsilon_{it}$$

(8) $$\;\;POL_{it}=\rho W_{ij}POL_{it}+\alpha_{1}ER_{it}+\alpha_{2}W_{ij}ER_{it}+\beta X_{it}+\varepsilon_{it}$$

(9) $$GIE_{it}=\rho W_{ij}GIE_{it}+{c^{\prime }}_{1}ER_{it}+{c^{\prime }}_{2}ER_{it}^{2}+bPOL_{it}+\lambda X_{it}+\theta_{1}W_{ij}ER_{it}+\theta_{2}W_{ij}ER_{jt}^{2}+\theta_{3}W_{ij}POL_{it}+\gamma W_{ij}X_{it}+\varepsilon_{it}$$

In Equations (8) and (9), $POL_{it}$ is the proportion of polluting industries, measured using the proportion of total assets of polluting industries to total assets of industrial enterprises above the scale. Coefficients $c_{1}$, $c_{2}$, $c_{3}$ and $c_{4}$ measure the impact of ER on GIE, and coefficients $\alpha_{1}$ and $\alpha_{2}$ measure the environmental regulations’ impact on the proportion of polluting industries. Coefficient b and coefficients $c_{1}^{\prime }$ and $c_{2}^{\prime }$ measure the effect of the proportion of polluting industries and environmental regulations on GIE, respectively, while other variables have the same meaning as those in Equation (4).

According to the test for mediating effects, if coefficients $c_{*}$, $\alpha_{*}$, b and $c_{*}^{\prime }$ are significant, then a partial mediating effect exists. If coefficients $c_{*}$, $\alpha_{*}$ and b are significant and coefficient $c_{*}^{’}$ is insignificant, then a full mediation effect exists. If coefficient $c_{*}$ is significant and at least one of the coefficients $\alpha_{*}$ and b is insignificant, a Sobel test is required to determine whether there is a mediating effect [48].

### 3.2. Variable Description

Explained variable: Green innovation efficiency (*GIE*). We measured Chinese provinces’ GIE using the nonradial, nonangular Super-SBM-DEA model. The R&D capital stock and full-time equivalent of R&D personnel were selected as green innovation inputs [49]. The number of domestic invention patent applications received and new product sales revenue were selected as the expected output of green innovation. Wastewater emissions, emissions and solid waste generation were selected as the non-desired outputs of green innovation [50].

Core explanatory variable: Environmental regulation (*ER*). There is a lack of uniformity in the measurement of environmental regulation. First, existing studies have chosen input indicators, such as pollution control costs, pollution tax revenues, and energy consumption, to measure environmental regulation [51,52]. Second, from the output perspective, environmental regulation is measured by using pollutant emissions or synthesizing them into a composite index [53,54]. Third, the GDP/Energy indicator proposed by Ben Kheder and Zugravu-Soilita [55] is used to measure environmental regulation, and they argue that the advantage of using this indicator is that it measures the real impact effect of environmental laws and conditions imposed by the government. Thus, some provinces only implement “theoretical” environmental regulation policies without any constraints on enterprises, while some provinces have adopted real environmental regulation policies for production enterprises. Therefore, this indicator is a good measure of the real effect of government environmental regulation.

Specifically, under the condition of constant GDP, the less energy consumption, the corresponding reduction in pollution emissions, the greater the value. This shows that the more obvious the effect of energy saving and emission reduction, it also indicates that the intensity of environmental regulation will be stronger. This shows that a more obvious effect of energy saving and emission reduction also indicates that the intensity of environmental regulation will be stronger. In view of the superiority of this indicator, this paper chooses GDP/Energy to measure the intensity of environmental regulation in 30 Chinese provinces. As this ratio increases, the degree of environmental regulation becomes more stringent. In addition, to test the robustness of the findings in this study, we also construct a composite index of pollutant emissions to characterize environmental regulations.

Control variables: Drawing on the research of Hao et al. [53] and Hong et al. [56], the control variables selected were foreign direct investment *(FDI*), financial support (*FIN*), industrial structure (*IND*), government R&D funding (*GOV*), economic development (*PGDP*) and human capital (*HC*), where foreign direct investment is evaluated by the amount of *FDI* as a proportion of regional GDP; financial support is evaluated by the proportion of internal expenditure on R&D funding that is loaned by financial institutions; government R&D funding is evaluated by the proportion of government funding within R&D expenditure; the industrial structure was measured using the secondary sector as a proportion of regional GDP; the level of PGDP was measured using GDP per capita, using the GDP per capita index, which was deflated using 2004 as the base period; and human capital is evaluated using the average number of years of schooling of the population.

### 3.3. Descriptive Statistics

Provincial-level panel statistics of China’s 30 provinces for the period 2004–2017 were used as the study sample (data limitations, excluding Tibet, Hong Kong, Macau and Taiwan), which are from the *China Statistical Yearbook*, *China Environmental Statistical Yearbook*, *China Statistical Yearbook on Science and Technology*, *China Population Statistics Yearbook* and provincial statistical yearbooks. The results of the descriptive statistics for the variables are shown in Table 1.

## 4. Empirical Results

### 4.1. Measurement and Analysis of GIE

Based on the selected input-output indicators, the Super-SBM-DEA method was applied to evaluate the GIE of 30 provinces in China from 2004 to 2017. Then, the average GIE of the whole country and the eastern, central and western regions was obtained. The results are shown in Table 2. During the inspection period, the national average value of green innovation efficiency was 0.4794, with 0.6762, 0.3928 and 0.3454 for the eastern, central and western regions, respectively. It is widely acknowledged that there is a large spatial variability in the GIE in different regions of China, with the highest efficiency values in the eastern region, and the difference in efficiency values between the eastern and central regions is markedly greater than that between the midwest. This indicates that the innovation mechanism in the eastern region is working well and green innovation is more dynamic [57]. In contrast, the innovation mechanism in the mid-west needs to be improved, and green innovation has failed to contribute to economic development as much as it should.

The possible reason for this is that the eastern region has a much higher level of economic development than the central region and has a relatively larger scale of green innovation investment [58], which implies a higher green innovation efficiency. The relative backwardness of economic development in the central region not only implies insufficient investment in innovation capital, but also further leads to technology brain drain [59]. Therefore, the central region lacks the intrinsic drivers of green innovation. In addition, a higher proportion of heavy industrial enterprises leads to more serious environmental pollution in the central region, which becomes one of the reasons for the lower GIE in the central region [60]. Compared to the central region, the western region not only has worse innovation conditions, such as insufficient innovation investment, brain drain and environmental pollution [61], but also has a bias to value economic growth at the expense of environmental protection. This may lead to short-sighted behaviors, such as sacrificing the environment to develop the economy in the western region for a long time [62]. As a result, the western region has the lowest green innovation efficiency.

### 4.2. Spatial Correlation Test and Model selection

Stata software was applied to estimate the Moran’s I of environmental regulation and GIE for the period of 2004–2017 (see Table 3). The Moran’s I of ER was significantly positive in all years of the period under consideration. The Moran’s I of GIE was also significantly positive in all years except 2004. This indicates that the two variables are not spatially randomly distributed but show a clear positive spatial correlation. Test results of model selection is shown in Table 4, and a spatial panel Durbin model considering time-space fixed effects was chosen for estimation, meanwhile, time-fixed effects model was also estimated for comparison.

### 4.3. Results of the “Local Neighborhood” Effect

Given that there is a significant positive spatial correlation between ER and GIE, ordinary panel regression models may lead to biased results by ignoring this spatial correlation. Therefore, first, a general panel regression model was applied to analyze the effect of ER on GIE without considering spatial factors (Table 5), and second, a spatial Durbin model was applied to analyze the “local neighborhood” effect (Table 6). To avoid possible endogeneity problems between ER and GIE, the first-order lagged term L.ER of ER is used as its instrumental variable. The previous year’s environmental regulation is correlated with the present year’s environmental regulation, but L.ER is a predetermined variable that is not correlated with the present year’s random error term and meets the definition of an instrumental variable.

In the non-spatial panel estimation, we mainly used the fixed effects model and the Tobit model. Traditional mixed regressions ignore unobserved heterogeneity among individuals that may be correlated with explanatory variables and lead to inconsistent estimates. In contrast, fixed effects model takes individual effects into account, assuming that individual regression equations have the same slope but can have different intercept terms, thus capturing heterogeneity and making the regression results more accurate. In addition, Tobin [63] pointed out that ordinary least squares (OLS) is no longer suitable for estimating regression coefficients when the dependent variable is a restricted variable, and then the Tobit model, which follows the concept of maximum likelihood estimate, is a better choice. Since the explanatory variable of this paper, GIE, is a restricted variable, the Tobit model is chosen for estimation in the non-spatial panel case.

First, the two-stage least squares method was applied to explore the impact of environmental regulation on GIE when spatial factors were not taken into account, and the results are shown in Table 5. As is known from Table 5, the first-stage regression coefficient values are markedly at the 1% level, with F values well above 10, indicating that L.ER is appropriate as an instrumental variable. In the second-stage regression, the effect of ER on GIE has a significant “U”-shaped relationship in both the panel fixed effects model and the panel Tobit model. Hypothesis 1 was tested. The panel fixed effects model estimated an inflection point value of 0.74, and the panel Tobit model estimated an inflection point value of 0.62.

Further incorporating spatial factors to analyze the “local-neighborhood” effect of *ER* on *GIE*, the *LR* test rejects the time-fixed effects model and, therefore, uses both a space fixed effects model and a time-space dual fixed effects model. The regression results are shown in Table 6. It is clear that, among the local effects, the influence of *ER* on the local *GIE* is first manifested as a disincentive and then as a facilitator, which further tested Hypothesis 1. The space fixed effects model estimated an inflection point value of 0.63, which is very similar to the inflection point value of 0.62 estimated by the panel Tobit model. The estimated inflection point value of the double fixed effects model is 0.57, which is lower than the estimated inflection point value of the panel fixed-effects model and the panel Tobit model. It shows that after controlling time and space at the same time, the inflection point of the “U” curve of the impact of *ER* on local *GIE* is shifted to the left, and it is easier for enterprises to succeed in a win-win situation between green innovation performance and *ER* [64]. The estimation results of the neighborhood effect show that the effect of ER on GIE in the neighborhood shows an inverted “U”-shaped feature of promotion followed by inhibition, and Hypothesis 2 was verified.

### 4.4. Results of the Transmission Channels of the Neighborhood Effect

Table 6 shows the estimation consequences of the regression on Equation (7). What can be found is that environmental regulation significantly affects GIE, i.e., the coefficient $c_{*}$ is significant, so it is necessary to further confirm whether the coefficients $\alpha_{*}$, *b* and $c_{*}^{’}$ are significant. A regression of Equation (8) was performed, and the results are shown in Table 7. It is widely acknowledged that the effect of *ER* on the proportion of local polluting industries is significantly negative, while the effect on the proportion of polluting industries in neighboring areas is significantly positive, with a significant coefficient $\alpha_{*}$ in both the space fixed effects model and the time-space dual fixed effects model. This suggests that ER policies reduce the share of local polluting industries and increase the share of polluting industries in neighboring areas, i.e., environmental regulation causes local polluting industries to move to neighboring areas.

We further introduce the proportion of polluting industries to Equation (9) to research the influence of the proportion of polluting industries and ER on the regional GIE. The regression results are shown in Table 8. The effect of the proportion of polluting industries on local GIE is significantly negative in both the space fixed effects model and the time-space dual fixed effects model, and the effect of ER on the local GIE is significant in a “U” shape. That is, coefficients b and $c_{*}^{\prime }$ are significant, and there is a partial mediation effect. This shows that ER has a significant influence on GIE in neighboring places through the transfer of polluting industries, in addition to its direct effect on local GIE.

### 4.5. Robustness Tests

To ensure the credibility of the research results, three robustness tests were performed: (1) The spatial weight matrix was replaced. The geographical adjacency matrix (W1) assumes that there is no connection between regions if they are not adjacent. This setting does not correspond to reality, so the geographic distance matrix W2 is constructed to re-estimate the model [21]. (2) A new environmental regulation index for regression from an output perspective was constructed. Zhou et al. [65] believed that a comprehensive index constructed from pollutant emissions can reflect the intensity of environmental regulation. Drawing on their ideas, a new environmental regulation indicator *ER1* was constructed using sulfur dioxide emissions, solid waste generation and wastewater emissions, and regression analysis was performed. In the evaluation system of environmental regulation indicators, GDP/Energy is measured in terms of the regulatory effect, while the pollutant emissions are considered in terms of pollution output. Scholars generally point out that in the short term, the higher the intensity of major pollutant emissions, the stricter the governmental regulation measures [66,67]. In the long run, the government has adopted strict environmental regulation policies so that high-polluting enterprises have to bear high environmental costs and try to reduce pollutant emissions [68]. In China, in both the short and long term, there is no doubt that the degree of environmental regulation by local governments is strongly correlated with local pollution emissions. In general, relatively good environmental quality and low pollutant emissions are associated with relatively low levels of environmental regulation. The pollutant emission data can reflect the government’s efforts on pollution control, and it can truly reflect the enforcement of environmental regulation. Therefore, the pollutant composite index can be used to evaluate environmental regulation. (3) The measurement method was changed. A threshold model was used to research the influence of ER on GIE.

The regression consequences of the first and second robustness tests are detailed in Table 9. After using different spatial weighting matrices and environmental regulation indicators, the obtained coefficients are inconsistent, but the results all support that the local effect of ER on GIE is “U”-shaped, and the neighborhood effect is an inverted “U”-shape, which indicates that the conclusion is robust and credible.

The results of the robustness tests for the third term are detailed in Table 10 and Table 11, and Table 10 shows that there is a single threshold effect for ER, which is significant at the 5% level. The threshold value is 0.6145, which is basically consistent with the inflection point of the “U” curve obtained by the panel Tobit model.

Further performing threshold regression analysis, Model (1) shows that environmental regulation has an inhibiting influence on local GIE when it is less than the threshold value, but it is not significant. After crossing the threshold value, ER has a significant promoting influence on GIE. Given that the coefficients on industrial structure and level of economic development are not significant, after removing these two control variables and rerunning the threshold effect test, the threshold value remains the same, and the *p* value is smaller. Model (2) shows the regression result. It is widely acknowledged that ER has a significant inhibiting effect on local GIE when it is less than the threshold value. After crossing the threshold value, *ER* has a significant promoting influence on local GIE; at the same time, the “U”-shaped curve holds, and the conclusion is robust.

## 5. Discussion

In the local effect, the effect of ER on the efficiency of local green innovation shows a “U”-shaped characteristic of first inhibiting and then promoting. That is, when the intensity of ER is low, stronger ER by the government will cause the cost of pollution control to rise for firms, leading to reduced profits and squeezing out R&D funds, thus inhibiting GIE [69]. When the intensity of ER is high, the pressure of excessive pollution abatement costs forces firms to increase R&D and green innovation to improve production technology and pollution abatement technology to form a competitive advantage [70], thus promoting green innovation efficiency. More studies support our conclusion. Popp et al. [71] found that because the cost increase brought by ER does indeed compress the profit margin of enterprises, and at the same time, enterprises lack the willingness to innovate in green technology, so ER will inhibit green innovation of enterprises in the short term. Regarding the “innovation compensation” effect of environmental regulation, Porter and van der Linde [72] argue that the innovation benefits of technological progress can exceed the costs of regulation, and, therefore, reasonable environmental regulation can effectively stimulate technological progress and innovation in the regulated firms. Acemoglu et al. [11] further classifies production sectors into clean and non-clean types and analyzes the impact of environmental policy incentives on technological innovation by constructing a direction of technological progress model. Their numerical simulation results find that some environmental regulatory measures, such as pollution taxation and R&D subsidy policies, can promote clean technological innovation without sacrificing economic growth.

Regarding the control variables, the effect of FDI on GIE is markedly positive, indicating that the large amount of capital, advanced technology, etc., it brings in has contributed to local green innovation efficiency [73]. The influence of financial support on GIE is significantly negative, indicating that banks have “adverse selection” to small, medium and micro high-tech enterprises when they issue loans, and when monitoring the use of loan funds, it will restrict the reinvestment of enterprises in R&D, resulting in a mismatch of financial resources [74], inhibiting the improvement of local GIE. The effects of government R&D funding, industry structure and level of economic development on green innovation efficiency are markedly positive in the space fixed effects model and positive but nonsignificant in the double fixed effects model. This illustrates that government R&D funding promotes local GIE through the provision of innovation funding and policy guidance [75]. An improved industrial structure facilitates the development of local GIE. The higher the level of PGDP, the better the supporting innovation mechanisms usually are, and the more conducive to GIE. The effect of HC on GIE is not significant in either model, and it is generally assumed that the accumulation of human capital will facilitate technological innovation [76]. Nevertheless, several scholars believe that the combination of human capital accumulation and rational allocation of human capital can promote technological innovation. Differences in the relative compensation structure of departments will cause a mismatch of human capital and even make human capital in the monopoly sector inhibit technological innovation.

In the neighborhood effect, the influence of ER on GIE in neighborhoods shows an inverted “U” shape of promotion followed by inhibition. When the intensity of local environmental regulations is high, local polluting industries will probably move to neighboring areas [77]. In the short term, the take-up of industries by neighboring regions will lead to an increase in output value and income levels, which, in turn, will increase R&D expenditures for innovation and promote green technology innovation in neighboring regions. However, in the long run, the neighboring land will become the undertaking place of high pollution and low technology industries. Its industrial structure will probably also change and develop in the direction of non-cleaning, deteriorating its ecological and industrial environment and inhibiting the green innovation efficiency of the neighboring regions. Some scholars have also focused on the phenomenon of transboundary pollution by ER. Wu et al. [78] found that since the promulgation of China’s Eleventh Five-Year Plan emission reduction mandate, firms began to migrate from coastal provinces with relatively stringent environmental regulations to the central and western regions. Li et al. [79] found that ER increased the probability of firm migration based on micro-firm data in China, but the paths and motivations of such migration differed significantly between pollution-intensive firms and clean firms. Dechezleprêtre et al. [80] found that increasingly stringent climate control policies in developed countries have contributed to a significant increase in the number of low-carbon patent applications in China.

Relating to control variables, the coefficients on FDI, financial support, level of economic development and human capital are all nonsignificant, indicating that their spillover effects on neighboring areas are limited. Government R&D funding significantly inhibits green innovation efficiency in neighboring areas in both models, indicating that government R&D funding guides the flow of innovation resources to local areas through policy signals and competes with neighboring areas, which is not advantageous to the improvement of GIE in neighboring areas [81]. The influence of industrial structure on GIE in neighboring areas is significantly negative in the double fixed effect model and negative but not significant in the space fixed effect model, which may be the transfer of polluting enterprises to neighboring areas in the adjustment of local industrial structure [82], which inhibits the improvement of GIE in neighboring areas.

## 6. Conclusions

According to the provincial-level panel statistics of Mainland China for the period 2004–2017, this paper employs the Super-SBM-DEA method to evaluate the *GIE* of China’s provinces and constructs a spatial Durbin model and a mediating effects model to analyze the “local-neighborhood” effect of ER on GIE. The study indicates that (1) there are large discrepancies in GIE between regions in China, with the eastern region having a much higher GIE than the midwest, the difference in efficiency values between the eastern and central regions being much higher than that between the central and western regions, and the distribution of GIE having a significant positive spatial correlation. (2) The local effect of ER on GIE has a “U” shape. The neighborhood effect of ER on GIE has an inverted “U” shape. (3) The neighborhood effect of ER on GIE is achieved through the transfer of local polluting industries.

## 7. Recommendations

Based on the above conclusions, this paper makes the following policy recommendations:

(1) Focus on regional differences and adopt innovative support policies according to local conditions. In response to the distribution pattern of GIE in China, which is “high in the east and low in the west”, the government should adopt different innovation support policies according to local conditions. The eastern region should focus on independent innovation, guide banks and other financial organizations to provide financial support to small and medium-sized high-tech companies; adjust the relative remuneration structure of the workforce, reduce financial mismatches and mismatches in human capital resources; and further smooth the innovation mechanism and play a demonstration and driving role. Increase government funding for R&D in the mid-west and use policy advantages to make up for regional disadvantages. Improve the green innovation environment in terms of tax incentives and R&D subsidies to attract high-quality investment and support the eastern regions in transferring suitable green technologies and green industries to the mid-west to help develop their green innovation capabilities.
(2) Tailor environmental regulation policies to local realities. The relationship between ER and the local GIE has a “U”-shaped feature. According to the current state of the local economy and natural resources, the government can reasonably adjust the intensity of environmental regulation so that the implementation of environmental regulation can achieve optimal results. Given that most of the regions that have not reached the inflection point are the less economically developed central and western regions, the government should take into account local economic development and employment levels and flexibly use a variety of environmental regulatory tools. For example, the government could use administrative environmental regulation tools to establish a bottom line, market-based environmental regulation tools to promote efficient resource allocation, and green innovation by local enterprises through government subsidies. For regions where the level of ER has crossed the inflection point, the government can enhance environmental regulation in conjunction with actual practice, such as strictly restricting pollutants as factor inputs, requiring the establishment of pollutant reduction equipment, encouraging media and public participation in environmental regulation and supervision, etc., motivating enterprises to further increase investment in green innovation and take the high-end development path of innovation-driven and green transformation.
(3) Integrating regional environmental regulation policies. In response to the problem that environmental regulation can cause polluting industries to move across regions, the central government should comprehensively consider the actual differences and interest appeals of various regions to form a cross-regional environmental collaborative governance system led by one party and participated by multiple parties to reduce polluters’ incentive to move across regions and to avoid local governments’ beggar-my-neighbor in environmental regulation issues. Local governments should strengthen collaboration and reach consensus in policy implementation and regulation to bring into play the spatial linkage of environmental regulation policies. In addition, it is necessary to refine and diversify environmental regulation policies, formulate specific regulatory measures for polluting and nonpolluting industries, appropriately increase the locational transfer costs of polluting industries, raise their market entry barriers, strictly rectify polluting enterprises with backward technologies within a time limit, and provide certain financial and technical policy support to assist and force them to carry out R&D investment and improve their production processes to abate pollution at the source.

## Figures and Tables

**Table 1:** Statistical description of variables.

Variable	Symbols	Obs	Mean	Std.Dev.	Min	Max
Green innovation efficiency	*GIE*	420	0.4794	0.2948	0.0299	1.2711
Environmental regulation	*ER*	420	0.5455	0.2352	0.1342	1.2480
Foreign direct investment	*FDI*	420	0.4000	0.5076	0.0473	5.7054
Financial support	*FIN*	420	0.0414	0.0270	0.0046	0.2331
Government R&D funding	*GOV*	420	0.2388	0.1263	0.0687	0.6081
Industrial structure	*IND*	420	0.4630	0.0798	0.1897	0.5905
Economic development	*PGDP*	420	0.4124	0.5084	−0.8400	1.8085
Human capital	*HC*	420	8.6811	1.0019	6.3778	12.6651

**Table 2:** GIE measurements in China, 2004–2017.

Year	National	Eastern	Central	Western
2004	0.4002	0.6516	0.2191	0.2807
2005	0.3889	0.6475	0.2657	0.2198
2006	0.4435	0.7440	0.2832	0.2596
2007	0.4306	0.7377	0.2690	0.2410
2008	0.3910	0.6634	0.2431	0.2261
2009	0.3496	0.5173	0.3282	0.1974
2010	0.4243	0.6391	0.3337	0.2753
2011	0.4935	0.7138	0.3847	0.3522
2012	0.5202	0.6669	0.4795	0.4031
2013	0.5465	0.7184	0.4560	0.4405
2014	0.5337	0.6569	0.4634	0.4617
2015	0.5310	0.6084	0.4989	0.4769
2016	0.6142	0.7409	0.6321	0.4744
2017	0.6439	0.7609	0.6431	0.5275
Mean	0.4794	0.6762	0.3928	0.3454

**Table 3:** The test results of *Moran’s I*.

Year	Environmental Regulation		Green Innovation Efficiency	
	*Moran’s I*	Z Value	*Moran’s I*	Z Value
2004	0.6110 ***	5.2230	0.1530	1.5360
2005	0.5950 ***	5.0960	0.3410 ***	3.1140
2006	0.5710 ***	4.9120	0.3870 ***	3.4750
2007	0.5500 ***	4.7590	0.4020 ***	3.6150
2008	0.5490 ***	4.7640	0.4570 ***	4.0620
2009	0.5430 ***	4.7390	0.2420 **	2.3680
2010	0.5160 ***	4.5330	0.4120 ***	3.6370
2011	0.4780 ***	4.2610	0.3840 ***	3.4050
2012	0.4660 ***	4.1770	0.4010 ***	3.5620
2013	0.4000 ***	3.7080	0.4580 ***	4.0290
2014	0.3900 ***	3.6300	0.3560 ***	3.1880
2015	0.3670 ***	3.4720	0.3010 ***	2.7450
2016	0.3580 ***	3.4210	0.3570 ***	3.1560
2017	0.3640 ***	3.4610	0.1750 *	1.7020

Note: *, ** and *** denote significance levels of 10%, 5% and 1%, respectively.

**Table 4:** Test results of model selection.

Test	W1		W2	
	Statistic	*p*-Value	Statistic	*p*-Value
LM-lag	18.413	0.000	88.717	0.000
Robust LM-lag	0.019	0.890	0.965	0.326
LM-error	104.936	0.000	156.530	0.000
Robust LM-error	86.542	0.000	68.778	0.000
LR-lag	24.66	0.002	18.68	0.017
LR-error	88.07	0.000	16.48	0.036
Wald-lag	34.02	0.000	22.12	0.005
Wald-error	57.42	0.000	21.21	0.007

**Table 5:** Baseline regression results.

Variables	Panel Fixed Effects Model	Panel Tobit Model
*ER*	−1.3446 ** (−3.0706)	−0.7261 ** (−2.4654)
*(ER)^2^*	0.9080 *** (3.3808)	0.5859 ** (2.7175)
*FDI*	0.1074 *** (4.4553)	0.1044 *** (4.4861)
*FIN*	−1.2371 ** (−3.2970)	−1.2870 *** (−3.5072)
*GOV*	−0.3538 * (−1.7972)	−0.3524 ** (−2.2148)
*IND*	0.1478 (0.6073)	−0.1624 (−0.9735)
*PGDP*	0.1038 (0.7550)	0.1850 ** (2.9962)
*HC*	0.1000 ** (2.9397)	0.0882 *** (7.4489)
*cons*	0.0039 (0.0100)	__
Regression of the first stage		
*L.ER*	0.9580 *** (158.2614)	0.9580 *** (158.2614)
*F*	25,046.68 ***	25,046.68 ***
*N*	390	390
*R^2^*	0.2965	—

Note: Values in parentheses are *t* values, *, ** and *** indicate significance at the 10%, 5% and 1% levels, respectively.

**Table 6:** The “local neighborhood” effect results.

Variables	Space Fixed Effects Model		Time-Space Double Fixed Effects Model	
	Local Effects	Neighborhood Effects	Local Effects	Neighborhood Effects
*ER*	−1.6412 ** (−3.2189)	0.5723 ** (2.5155)	−1.3179 ** (−2.3460)	0.5016 * (1.9365)
*(ER)^2^*	1.2993 *** (3.8830)	−0.3593 ** (−2.5336)	1.1521 *** (3.3387)	−0.3112 ** (−2.0850)
*FDI*	0.0737 ** (2.9471)	−0.0540 (−1.3071)	0.0785 ** (3.1034)	−0.0148 (−0.2889)
*FIN*	−0.9096 ** (−2.3126)	−0.0853 (−0.3862)	−1.0621 ** (−2.6685)	−0.1737 (−0.7085)
*GOV*	0.3631 * (1.6949)	−0.2140 ** (−2.0057)	0.3820 (1.6136)	−0.2650 ** (−2.1655)
*IND*	0.5721 ** (2.1590)	−0.1454 (−1.2391)	0.4785 (1.3629)	−0.4227 ** (−2.9306)
*PGDP*	0.3831 ** (2.3671)	−0.0862 (−1.2490)	0.3174 (1.4410)	0.0044 (0.0580)
*HC*	0.0067 (0.1646)	−0.0108 (−0.7794)	−0.0132 (−0.2576)	0.0027 (0.1515)
*Spatial-ρ*	0.2791 *** (42.9799)		0.2803 *** (41.7252)	
*Log-L*	139.8728		147.1556	
*N*	390		390	
*R^2^*	0.1267		0.0234	

Note: Values in parentheses are *t* values, *, ** and *** indicate significance at the 10%, 5% and 1% levels, respectively.

**Table 7:** Consequences of the “local neighborhood” effect of ER on polluting industries.

Variables	Space Fixed Effects Model		Time-Space Double Fixed Effects Model	
	Local Effects	Neighborhood Effects	Local Effects	Neighborhood Effects
*ER*	−0.2835 *** (−7.7175)	0.0661 *** (3.6605)	−0.3425 *** (−7.7671)	0.0685 *** (3.0580)
*FDI*	0.0087 (1.5381)	0.0182 ** (1.9828)	0.0072 (1.2353)	0.0074 (0.6411)
*FIN*	−0.3083 *** (−3.4489)	0.0651 (1.3096)	−0.2855 *** (−3.1114)	0.1160 ** (2.0612)
*GOV*	0.0121 (0.2506)	0.0175 (0.7341)	0.0472 (0.8667)	0.0225 (0.7990)
*IND*	0.0108 (0.1851)	0.0052 (0.2092)	−0.0131 (−0.1668)	0.0622 * (1.9485)
*PGDP*	−0.0708 * (−1.9322)	0.0303 ** (2.1527)	−0.0089 (−0.1769)	0.0066 (0.4088)
*HC*	−0.0407 *** (−4.4351)	0.0123 *** (3.9251)	−0.0305 ** (−2.5700)	0.0140 *** (3.3769)
*Spatial-ρ*	0.2799 *** (42.8420)		0.2807 *** (41.9825)	
*Log-L*	717.2349		718.8258	
*N*	390		390	
*R^2^*	0.3074		0.2021	

Note: Values in parentheses are *t* values, *, ** and *** indicate significance at the 10%, 5% and 1% levels, respectively.

**Table 8:** The mechanism results of the neighborhood effect.

Variables	Space Fixed Effects Model	Time-Space Double Fixed Effects Model
	Local Effects	Local Effects
*ER*	−2.0078 *** (−3.7093)	−1.7644 *** (−2.8167)
*(ER)^2^*	1.4613 *** (4.2554)	1.3417 *** (3.6853)
*POL*	−0.4689 * (−1.8914)	−0.4652 * (−1.8015)
*FDI*	0.0766 *** (3.0774)	0.0817 *** (3.2449)
*FIN*	−1.0613 *** (−2.6753)	−1.1915 *** (−2.9727)
*GOV*	0.3670 * (1.7199)	0.4149 * (1.6874)
*IND*	0.5573 ** (2.0900)	0.4290 (1.2255)
*PGDP*	0.3475 ** (2.1424)	0.3277 (1.4671)
*HC*	−0.0178 (−0.4231)	−0.0269 (−0.5177)
*Spatial-ρ*	0.2791 *** (43.0051)	0.2800 *** (41.9149)
*Log-L*	142.3532	149.1165
*N*	390	390
*R^2^*	0.1449	0.0361

Note: Values in parentheses are *t* values, *, ** and *** indicate significance at the 10%, 5% and 1% levels, respectively.

**Table 9:** Robustness test results.

Variables	W2		W1		W2	
	Local Effects	Neighborhood Effects	Local Effects	Neighborhood Effects	Local Effects	Neighborhood Effects
*ER*	−0.6556 (−1.3631)	0.7625 (0.3829)				
*(ER)^2^*	0.6628 ** (2.2513)	−0.7049 (−0.6721)				
*ER1*			−0.2419 *** (−4.3650)	0.0774 ** (2.4911)	−0.1674 *** (−3.4740)	0.6633 *** (2.9760)
*(ER1)^2^*			0.0327 *** (3.2734)	−0.0072 (−1.1190)	0.0239 *** (2.7043)	−0.0884 ** (−2.1287)
*FDI*	0.0961 *** (4.4036)	0.0575 (0.2971)	0.0909 *** (3.8050)	0.0012 (0.0261)	0.0925 *** (4.4144)	−0.0455 (−0.2606)
*FIN*	−1.5157 *** (−4.4750)	−3.2728 *** (−2.8063)	−1.1472 *** (−2.9750)	0.2120 (0.9370)	−1.3271 *** (−4.0591)	−2.6836 ** (−2.3964)
*GOV*	−0.1107 (−0.6026)	0.1154 (0.1642)	0.2399 (1.0217)	−0.2616 ** (−2.1813)	−0.1620 (−0.8814)	0.0546 (0.0786)
*IND*	0.8245 *** (2.6620)	−0.8945 (−0.7691)	0.2987 (0.8324)	−0.1275 (−0.8772)	0.6365 * (1.9396)	1.2063 (0.9078)
*PGDP*	−0.0120 (−0.0624)	1.4155 ** (2.5078)	0.1282 (0.6151)	0.0067 (0.0981)	0.0298 (0.1690)	1.1240 ** (2.0768)
*HC*	−0.0394 (−0.8597)	−0.2541 (−1.6326)	−0.0218 (−0.4365)	0.0065 (0.3857)	−0.0179 (−0.3955)	−0.1755 (−1.1279)
*Spatial-ρ*	−0.3296 *** (−2.6797)		0.2812 *** (41.1612)		−0.2010 * (−1.6508)	
*Log-L*	254.0455		157.6752		263.4351	
*N*	390		390		390	
*R^2^*	0.0900		0.0013		0.0223	

Note: Values in parentheses are *t* values, *, ** and *** indicate significance at the 10%, 5% and 1% levels, respectively.

**Table 10:** Threshold effect test and threshold values.

Threshold Variables	Type of Threshold	Threshold Value	*p* Value	F Value
Environmental regulation	Single threshold	0.6145	0.0460	26.8200
	Double Threshold	0.4797	0.5320	10.9400
	Threefold threshold	0.4752	0.2000	15.8800

**Table 11:** The consequences of the threshold model.

	Model (1)	Model (2)
*ER(0 < ER* *≤* *0.6145)*	−0.1134 (−1.2700)	−0.1467 * (−1.6900)
*ER(ER > 0.614)*	0.2240 * (1.8600)	0.2022 * (1.7300)
*FDI*	0.1049 *** (4.5000)	0.1030 *** (4.4400)
*FIN*	−1.0655 *** (−2.9400)	−1.1294 *** (−3.1300)
*GOV*	−0.3487 * (−1.8000)	−0.3396 * (−1.7700)
*IND*	0.1521 (0.6200)	
*PGDP*	0.0456 (0.3400)	
*HC*	0.0964 *** (2.8700)	0.1108 *** (4.1000)
*POL*	−0.2744 ** (−2.5500)	−0.2818 *** (−2.6900)
*cons*	−0.6073 * (−1.8800)	−0.6702 *** (−3.4900)
*R^2^*	0.3315	0.3366

Note: Values in parentheses are *t* values, *, ** and *** indicate significance at the 10%, 5% and 1% levels, respectively.

## Data Availability

Not applicable.
